# Target marketing in the health services industry: the value of journeying off the beaten path

**DOI:** 10.1186/s12913-018-3678-5

**Published:** 2018-12-14

**Authors:** James K. Elrod, John L. Fortenberry

**Affiliations:** 1 Willis-Knighton Health System, 2600 Greenwood Road, Shreveport, LA 71103 USA; 2 LSU Shreveport, 1 University Place, Shreveport, LA 71115 USA

**Keywords:** Target marketing, Segmentation, Targeting, Positioning, Hospitals, Healthcare

## Abstract

**Background:**

Target marketing, a practice used to more effectively address the wants and needs of customers, involves three interrelated activities: market segmentation, targeting, and product positioning. The practice follows a perfectly logical process. For a given offering, healthcare institutions select a desired group to pursue and arrange service characteristics and related attributes in a manner to entice that particular group to forward patronage and become customers. Pursuits often focus on heavily-traveled routes teeming with competitors, but occasionally an off the beaten path can be identified to amplify target marketing efforts.

**Discussion:**

In an earlier chapter of its history, Willis-Knighton Health System identified and pursued an off the beaten path in its bid to capture market share in pediatric healthcare services. The direct route—targeting current and prospective parents—was heavily pursued by competitors, prompting the institution to seek a unique approach; a road less traveled which would reach the same audiences but do so via a different route. Children, as direct care recipients, supplied one such route, and while their ability to influence associated parental decisions was unclear, the institution viewed developing a bond with them to have great potential. Painstaking efforts yielded Willis-Knighton Health System’s Pediatric Orientation Program, fostering an affinity between the institution and children, which in turn influenced parents, affording opportunities for enhanced patronage in pediatric medicine and beyond.

**Conclusions:**

Willis-Knighton Health System’s decision to look off the beaten path for an avenue capable of amplifying its target marketing initiatives resulted in a novel pursuit which distinguished the institution from its competitors and set the stage for achieving its goal of providing healthcare services for a greater percentage of children in the marketplace. Additional spillover effects bolstering share in other areas also were afforded. This unique initiative addressed desires to pursue an increasingly important road less traveled to reach prime audiences. When roads less traveled can be identified, opportunities abound for better connecting with customer groups, warranting investigation and pursuit.

## Background

Healthcare establishments, especially comprehensive medical centers, offer myriad health and wellness services, affording communities with access to a wide array of offerings that address injury and illness [1, 2]. By providing such broad collections of services, health and medical institutions can make a case for employing a mass marketing approach to advance their organizations [3, 4]. With such an approach, available offerings are marketed broadly to the populace without regard for individual tastes and preferences [3, 5, 6]. These appeals are general in nature, designed to address the masses as a whole, hence the name mass marketing. Such one-size-fits-all strategies have their place, but very often, marketing results can be improved by engaging in what is known as target marketing, an approach which tailors services and their associated attributes to appeal to particular audiences sharing like characteristics and qualities [3, 6–9].

Emerging out of desires to more appropriately address the various wants and needs of different customer groups, target marketing involves three interrelated activities: market segmentation (i.e., dividing a market into groups exhibiting common characteristics), targeting (i.e., selecting attractive segments on which to focus), and product positioning (i.e., assembling service-related attributes in a manner to entice targeted audiences to extend their patronage) [3, 10]. The customized approach resulting from target marketing tends to resonate more powerfully with audiences than that afforded by mass marketing, permitting greater opportunities to convert prospects into customers [3, 4, 10]. Further, it improves customer satisfaction and also allows for better use of promotions resources by directing tailored communications to desired populations, minimizing wasted circulation [3, 4].

Target marketing makes sense; the practice follows a perfectly logical process. For a given offering, healthcare institutions select a desired group to pursue and arrange service characteristics and related attributes in a manner to entice that particular group to forward patronage and become customers. In many cases, health and medical services have fairly obvious targets. Women of childbearing age, for example, have potential needs for maternity services. Parents, courtesy of their infants and young children, have needs for pediatric medical care. Employers, due to their workforces, have needs for occupational health services. By focusing on the specific wants and needs of market segments, healthcare institutions can deliver services and support specifically designed and suited for the associated groups [3, 4].

Pathways leading directly to obvious target audiences for given offerings, however, are typically heavily traversed, with associated routes teeming with competitors eager to gain the affections and associated patronage of the designated groups. In essence, many establishments are using the same approaches directed toward the same audiences [11–15]. Such activity limits opportunities for market share gains, especially for those institutions not in market leadership positions, as the dominance of more powerful parties generally affords advantages over contender entities pursuing parallel strategies. In such cases, it is helpful to explore possible alternatives; roads less traveled that lead to the same target audiences, but do so via an indirect route, amplifying efforts to engage and attract desired groups [4, 11, 14].

With market competitiveness in the healthcare industry at all-time highs [3, 16–18], roads less traveled (i.e., novel approaches for addressing circumstances and situations which are known to and used by few, if any, rivals) are highly desirable, as they provide institutions with opportunities to differentiate themselves from competitors, increasing the likelihood of success in attracting prospects and encouraging exchange [3, 11–14]. Target marketing researchers in recent years have blazed new trails which reveal advantages associated with pursuing opportunities off the beaten path. For example, Kim and Mauborgne, in *Blue Ocean Strategy*, communicated the value of looking beyond the boundaries of established markets, directing attention toward uncontested market space for growth opportunities [3, 10–13]. Kotler and Trias de Bes, in *Lateral Marketing*, recommended that markets be viewed broadly in an effort to identify opportunities to serve customer groups that have previously been overlooked [3, 10, 14]. These perspectives essentially are suggesting that institutions forgo “follow the herd” mentalities or at least complement these pursuits with opportunities which set a new course and direction on roads less traveled. In similar fashion and illustrative of these modern thoughts on target marketing, Willis-Knighton Health System, in an earlier period of its history, discovered and followed a road less traveled in pursuit of a particular market share goal in a most challenging environment [19].

### Willis-Knighton Health System and market share growth ambitions

Willis-Knighton Health System is a nongovernmental, not-for-profit healthcare provider delivering comprehensive health and wellness services through multiple hospitals, numerous general and specialty medical clinics, an all-inclusive retirement community, and more. Based in Shreveport, Louisiana, the system holds market leadership in its served region, centered in the heart of an area known as the Ark-La-Tex, where the states of Arkansas, Louisiana, and Texas converge. Willis-Knighton Health System’s extensive service array can accommodate virtually any medical care want or need, regardless of one’s age. This, combined with its market leadership, attracts patient populations from across the region, fueled further by the institution’s acceptance of most health insurances and, notably, its provision of substantial amounts of charity care for those unable to pay for services. The system’s origins date to 1924 with the establishment of Tri-State Sanitarium, founded to address the healthcare needs of the burgeoning population of west Shreveport. Sold in 1929 to Drs. James Willis and Joseph Knighton, the establishment continued operations and, in 1952, it was renamed in honor of Drs. Willis and Knighton. For the first several decades of its existence, the establishment played an important but relatively small role in delivering the region’s healthcare. In the 1970s, however, Willis-Knighton Health System embarked on an ambitious growth campaign to expand its footprint beyond west Shreveport, effecting a number of strategies [20–22], notably including pursuit of market share in the ultra-competitive area of pediatric medical services.

During this period, the greater Shreveport marketplace was burgeoning with young families, making pediatric care a very lucrative service line for healthcare establishments capable of attracting associated patronage. In the area of labor and delivery services, the gateway to provision of years of pediatric care as newborns grow older, Willis-Knighton Health System possessed less than 10% of the available share in the market [19]. Share improvements were needed, especially as success in this area would likely generate spillover effects with the potential to bolster share in other areas. Achieving this, however, would be anything but easy, as the market featured a number of competitors, including one possessing market leadership in pediatrics and virtually all other categories of care. Competitive assessments revealed that all were targeting current and prospective parents—the direct and obvious target market—in their bids to maintain or grow market share in pediatric medicine. Realizing that the upside potential associated with mirroring competitive approaches would be limited—something confirmed by the institution’s own prior efforts to build market share using like practices—executives decided to search for a unique approach; a road less traveled which would reach the same audiences but do so via a different route. The intention was for this to complement, rather than replace, Willis-Knighton Health System’s existing target marketing efforts directed toward current and prospective parents.

A comprehensive evaluation of the pediatric healthcare patronage process ensued. As evidenced by the target marketing pursuits of competitors, parents represented the obvious target for pediatric medical services, as they occupied the all-important role of decider. Once parents had selected providers, children certainly played a role in customer retention, but in the context of target marketing, the operative question concerned their ability to influence the initial patronage decisions of their parents. There certainly was evidence from other industries that confirmed the influencing capability of children on the purchase decisions of parents [23–27], with retail (e.g., toys, foods) supplying perhaps the best example of this. Assuming that an affinity between institution and child could be nurtured, executives believed that the same would be observed in the area of healthcare services. Willis-Knighton Health System possessed a long-standing commitment to education, regularly holding seminars focused on things such as healthy eating, physical fitness, smoking cessation, and similar initiatives, prompting the notion that something in the area of children’s health education could provide an avenue for developing a bond with youth which, in turn, would influence their parents. Painstaking efforts yielded Willis-Knighton Health System’s Pediatric Orientation Program.

### Willis-Knighton Health System’s Pediatric Orientation Program

Initiated in 1979 and now in its 39th year of operation, Willis-Knighton Health System’s Pediatric Orientation Program introduces first graders to hospitals and healthcare via an onsite tour and orientation session conducted at one of Willis-Knighton Health System’s institutions. Available to first grade classes at all schools in Caddo and Bossier parishes, students, accompanied by their teachers, are bused from their learning institutions to designated hospitals where they engage in a variety of health education activities. These informative field trips offer first graders the opportunity to learn about hospital operations, the roles played by various healthcare professionals, and the process of healthcare delivery from admission to discharge, all in a manner easily understood by young children. First graders are typically 6–7 years old and are embarking on their first year of elementary school, making for an excellent time to introduce them to matters of health and wellness. Among other learning experiences, students engage in role playing exercises—presenting as physicians, nurses, and patients, dressing in costumes fitting given roles—where they address health events which they themselves might encounter, such as a broken arm or tonsillitis. They also learn about healthy habits, including proper hygiene, nutrition, and physical fitness. Overall, the learning experience helps to acclimate students to hospitals and healthcare experiences, reducing or eliminating associated fears and allowing them greater comfort when facing their own illnesses and injuries or those experienced by family members and friends. Each child completing the orientation session receives a stethoscope and a hat featuring the Willis-Knighton Health System logo and the designation “Future Nurse” or “Future Doctor.”

For the educational benefit alone, Willis-Knighton Health System’s Pediatric Orientation Program was well worth its development, implementation, and investment. Over the course of its 39-year history, thousands of first graders have come to understand that hospitals and healthcare providers are there to benefit them, reducing fear and anxiety that otherwise might persist and negatively impact their willingness to welcome receipt of care. But the benefit of the program extends also to target marketing, something which initially prompted its development. Through these orientation sessions and the positive experiences that they provide, children gain familiarity with Willis-Knighton Health System. Such positive exposures have long-term benefits, as these children will eventually grow into adults with families of their own and associated healthcare needs, with these formative experiences likely influencing provider selections. In the nearer term, however, the parents of children completing the orientation program also gain exposure to the institution, indirectly but powerfully, potentially impacting their current pediatric care selections and possibly even influencing selections in other areas of care, including those pertaining to their own medical needs.

Parents must be informed of field trips and grant permission for their children to partake in them, bringing Willis-Knighton Health System to their attention as they consider and sign approval forms. Further, Willis-Knighton Health System invites parents to accompany their children during the Pediatric Orientation Program, providing another exposure opportunity. And even if parents choose not to attend, following the session, their children undoubtedly will share associated experiences with their mothers and fathers, offering yet another opportunity for parents to learn about Willis-Knighton Health System. Going forward, the children potentially will reflect on their enjoyable Willis-Knighton Health System experiences when matters of health and wellness come up, supplying additional opportunities for the institution to enter family discussions.

Essentially, through children and their experiences attending Willis-Knighton Health System’s Pediatric Orientation Program, parents gain exposure to the institution, opening the door for patronage consideration. Paired with target marketing initiatives that directly appeal to parents—the pathway traversed by most every competitor—this program added a road less traveled, creating dual streams of influence aimed at patronage deciders. Over time, this target marketing innovation, combined with other initiatives aimed at increasing pediatric medicine market share (e.g., recruitment of renowned pediatricians, construction of kid-friendly servicescapes, initiation of unique branding initiatives), resulted in Willis-Knighton Health System’s acquisition of market leadership in the category. Successes on the pediatric medicine front presented opportunities for share gains on other fronts, eventually leading the institution to market leadership in the region [19]. While this growth cannot be solely attributed to the innovative target marketing strategy introduced in the late 1970s, Willis-Knighton Health System’s Pediatric Orientation Program is believed to have played a meaningful role in the advancements achieved. From its origins to present day, this unique target marketing approach has not been copied by any of Willis-Knighton Health System’s competitors, creating a lasting competitive advantage.

## Conclusions

Willis-Knighton Health System’s decision to look off the beaten path for an avenue capable of amplifying its target marketing initiatives seeking pediatric medicine market share gains resulted in a novel pursuit—its Pediatric Orientation Program—which distinguished the institution from its competitors and set the stage for achieving its goal of providing healthcare services for a greater percentage of children in the marketplace. Additional spillover effects bolstering share in other areas also were afforded. This unique initiative addressed desires to pursue an increasingly important road less traveled to reach prime audiences, but beyond target marketing matters, it also afforded opportunities to address in positive fashion the health education needs of children in the region. Unique routes off the beaten path are not always available, but when they can be identified, opportunities abound for better connecting with desired audiences, courtesy of resulting energized target marketing efforts. Given associated benefits, healthcare institutions in search of enhancing market share would do well to actively explore target marketing possibilities situated off the beaten path.
